# Cas14a1-Mediated Nucleic Acid Diagnostics for Spinal Muscular Atrophy

**DOI:** 10.3390/bios12050268

**Published:** 2022-04-23

**Authors:** Zhiqing Hu, Miaomiao Chen, Chunhua Zhang, Zhuo Li, Mai Feng, Lingqian Wu, Miaojin Zhou, Desheng Liang

**Affiliations:** Center for Medical Genetics & Hunan Key Laboratory of Medical Genetics, School of Life Sciences, Central South University, Changsha 410078, China; huzhiqing@sklmg.edu.cn (Z.H.); chenmiaomiao@sklmg.edu.cn (M.C.); zhangchunhua@sklmg.edu.cn (C.Z.); lizhuo@sklmg.edu.cn (Z.L.); fengmai@sklmg.edu.cn (M.F.); wulingqian@sklmg.edu.cn (L.W.)

**Keywords:** CRISPR/Cas14a1, nucleic acid detection, asymmetric PCR, spinal muscular atrophy

## Abstract

Spinal muscular atrophy (SMA) is the main genetic cause of infant death. In >95% of the patients with SMA, the disease is caused by a single hotspot pathogenic mutation: homozygous deletion of exon 7 of the survival motor neuron 1 gene (*SMN1*). Recently, clustered regularly interspaced short palindromic repeats (CRISPR)/CRISPR associated protein (Cas)-based assays have been developed as a promising new option for nucleic acid detection. Here, we developed a Cas14a1-based assay combined with asymmetric PCR to establish a method for detection of the homozygous deletion of *SMN1* exon 7 in SMA patients. The minimum detectable concentration of genomic DNA reached 5.26 aM with our method, and the assessment of its detection performance in 33 clinical samples revealed that the results were completely consistent with those of multiple ligation-dependent probe amplification and quantitative PCR. Thus, our novel nucleic acid diagnostics combining CRISPR/Cas14a1 and asymmetric PCR not only provides specific and sensitive testing of the deletion of *SMN1* exon 7, but also holds promise for an accurate detection platform of genetic diseases and pathogens in multiple sample types.

## 1. Introduction

Spinal muscular atrophy (SMA) is one of the most common autosomal recessive inherited diseases in infancy, with an incidence of 1/6000–1/10,000. SMA is caused by mutations in survival motor neuron 1 gene (*SMN1*) that leads to irreversible degeneration of motor neurons in the spinal cord, and SMA patients experience progressive muscle weakness, difficulty swallowing, and eventually respiratory failure [1]. In >95% of SMA patients, the disease is caused by the following hotpot pathogenic mutation: homozygous deletion of *SMN1* exon 7 [2]. Moreover, a paralogous gene of *SMN1* exists, named *SMN2*, whose coding sequence differs from that of *SMN1* by a single base, the 6th base in exon 7 (T in *SMN2*, C in *SMN1*); however, *SMN2* undergoes alternative splicing and produces a truncated protein that cannot function normally [3].

Recently, Spinraza, Risdiplam and Onasemnogene abeparvovec have been approved for the treatment of SMA, and early therapy has been found to maximize the treatment benefit [4,5,6,7,8,9,10]. Therefore, accurate genetic diagnosis of SMA can facilitate early detection, intervention, and early treatment of the disease in patients, as well as prevent childbirth at the preimplantation stage.

For diagnosing SMA, several detection techniques have been developed, such as PCR-restriction fragment length polymorphism (PCR-RFLP) [11], quantitative PCR (qPCR) [12], multiple ligation-dependent probe amplification (MLPA) [13,14], Next generation sequencing [2], and droplet digital PCR [15]. These currently used detection methods differ in principle, scope of application, detection performance, and cost, but they all require bulky equipment and trained operators, which hinders the widespread application of the methods.

As a promising alternative approach for diagnosing infectious diseases, a nucleic acid detection strategy has recently been developed based on the clustered regularly interspaced short palindrome repeats (CRISPR)/CRISPR-associated protein (Cas) system; the detection depends on measuring the collateral cleavage of a nonspecific single-stranded DNA (ssDNA) or RNA reporter after the Cas protein specifically recognizes and cleaves the target DNA/RNA under the guidance of a CRISPR RNA (crRNA). Effector Cas proteins include Cas12, Cas13, and Cas14, and various Cas-based detection methods have been established, such as Cas13a-based SHERLOCK [16,17] and Cas12a-based DETECTR [18,19]. Previously, we used Cas12a for genetic testing of SMA and found that Cas12a was nonspecifically activated by *SMN2* in SMA patients when a wild-type *SMN1* crRNA was used. To eliminate the interference by *SMN2*, an artificial mismatch adjacent to c.840 was introduced into the *SMN1* crRNA to obtain a relatively more discernable difference between SMA patients and non-SMA individuals [20].

Cas14a is another member of the Cas family and is the smallest Cas protein reported thus far. Cas14a can recognize and cleave the target ssDNA and can be activated under crRNA guidance in the absence of a protospacer adjacent motif (PAM) sequence, a characteristic completely different from that of other Cas effector proteins [21,22,23]. Notably, Cas14a1 exhibits high sensitivity and specificity: Cas14a1 can distinguish between single-base differences efficiently without the requirement of artificially introduced mismatches, shows no PAM-sequence-related restriction, and can specifically detect any ssDNA that is complementary to the crRNA used. However, Cas14a has not been previously used for detecting human genetic diseases.

Here, we developed a Cas14a1-based nucleic acid detection method for a human genetic disease. Specifically, we integrated Cas14a1-based nucleic acid detection with asymmetric PCR to establish a method that enables rapid and cost-effective detection of homozygous deletion of *SMN1* exon 7 in SMA patients.

## 2. Materials and Methods

### 2.1. Clinical Samples

Deidentified samples were obtained from Hunan Jiahui Genetics Hospital. All sample donors signed an informed consent form. This study was approved by the Ethics Committee of the School of Life Sciences, Central South University (No. 2019-1-27).

### 2.2. Cas14a1 Protein Expression and Purification

The vector pLBH559_Tet-HisCas14a1Locus was a gift from Dr. Jennifer Doudna (Addgene plasmid #112502; http://n2t.net/addgene:112502 (accessed on 10 March 2021); RRID: Addgene_112502) [21,24]. The Cas14a1 fragment from this vector was ligated into pET28a expression plasmid to construct pET28a-Cas14a1, which was amplified by transformation into Escherichia coli strain DE3 (TransGen Biotech, Beijing, China). When the culture OD600 reached 0.8, isopropyl-β-d-thiogalactopyranoside (IPTG) (Sangon Biotech, Shanghai, China) was added into the culture medium at a 0.2 mM final concentration, and the bacterial cells were cultured for another 16 h with shaking at 15 °C. The bacteria were collected and lysed ultrasonically in a lysis buffer (50 mM Tris, pH 8.0, 300 mM NaCl, 20 mM imidazole, 1% Triton X-100, 1 mM DTT, and 1 mM PMSF), and after the affinity was chromatography performed using Ni-IDA columns equilibrated with 50 mM Tris, pH 8.0, 300 mM NaCl, and 20 mM imidazole buffer (Sangon Biotech), the target protein was eluted with equilibrium buffers of different concentrations of imidazole. Lastly, purified Cas14a1 protein was obtained after dialysis into a storage buffer (20 mM HEPES, pH 7.5, 1250 mM NaCl, and 10% glycerol) and filtering (pore size: 0.22 μm) and was stored at −80 °C.

### 2.3. crRNA Preparation

For constructing the crRNA expression plasmid, the pUC57-crRNA plasmid containing the T7 promoter was synthesized (Sangon Biotech). First, an in vitro transcription template was obtained through PCR performed using the primers crRNA-F and crRNA-SMN1-R (Sangon Biotech); next, the template was purified using a FastPure Gel DNA Extraction Mini Kit (Vazyme, Nanjing, China) and transcribed using a HiScribe^TM^ T7 High Yield RNA Synthesis Kit (New England BioLabs, Ipswich, MA, USA) at 37 °C for 16 h to prepare the crRNA; and, in the final step, the crRNA was purified using an miRNeasy^®^ Mini Kit (QIAGEN, Duesseldorf, Germany). The crRNA sequences and PCR primers used in this study are shown in Table S1 and Figure S1.

### 2.4. DNA Amplification

The genomic DNA (gDNA) was extracted from peripheral blood using the conventional phenol—chloroform method. About 2 µg gDNA was extracted from 200 μL of peripheral blood. PCR primers F1/R1 were designed to amplify exon 7 of human *SMN1* (NM_022874.2) and *SMN2* (NM_017411), and three types of PCR method were used in this study as follows: (1) PCR amplification, (2) secondary PCR, (3) asymmetric PCR. The PCR amplification was used to provide PCR products of induced pluripotent stem cells (iPSCs) derived from an SMA patient (SMA-iPSCs) and from an iPSC line derived from a non-SMA individual (N-iPSCs) for Sanger sequencing. The secondary PCR was used to produce ssDNA template for the SMA-Cas14a1 fluorescence detection system. Asymmetric PCR is another method for ssDNA template production, which can produce an ssDNA template more easily and rapidly. Secondary PCR was conducted in two steps as follows: First, traditional PCR was performed using F1/R1, with the 20 μL reaction volume containing 10 μL of 2× Phanta Max Master Mix (Vazyme), 7 μL of ddH_2_O, 1 μL of primer F1 (10 μM), 1 μL of primer R1 (10 μM), and 1 μL of genomic DNA (gDNA; 2 ng/μL); the thermocycling conditions were 94 °C for 5 min, followed by 35 cycles of 98 °C for 15 s, 57 °C for 15 s, and 72 °C for 15 s, and then elongation at 72 °C for 5 min. Next, 1 μL of the product from the first PCR was used as the template in the second PCR, where the reaction mixture contained 10 μL of 2× Phanta Max Master Mix, 8 μL of ddH_2_O, and 1 μL of primer F1 (10 μM), and the amplification protocol used was the same as that in the first PCR. In the case of asymmetric PCR, the 20 μL reaction volume contained 10 μL of 2× Phanta Max Master Mix, 3 μL of ddH_2_O, 5 μL of primer F1 (10 μM), 1 μL of primer R1 (1 μM), and 1 μL of gDNA (2 ng/μL); the thermocycling conditions were 94 °C for 5 min, followed by 50 cycles of 98 °C for 15 s, 57 °C for 15 s, and 72 °C for 15 s, and then elongation at 72 °C for 5 min.

### 2.5. SMA-Cas14a1 Assay Employing a Fluorescence Probe

Each SMA-Cas14a1 assay mixture contained 2 μL of 10× Cutsmart buffer (New England Biolabs), 100 nM Cas14a1, 125 nM crRNA, 300 nM fluorophore-quencher (FQ) probe (Sangon Biotech), 1 μL of PCR products, and nuclease-free water to a 20 μL total volume. The reaction solution was incubated at 37 °C for 60 min and the FAM fluorescence signal was measured every minute.

To determine the minimum gDNA concentration for distinguishing SMA patients and non-SMA individuals, DNA samples from both groups of study participants were serially diluted to 2, 1, 0.5, 0.1, 0.05, 0.01, and 0.005 ng/µL and were tested using the SMA-Cas14a1 assay.

### 2.6. Statistical Analysis

Fluorescence signals were statistically analyzed using GraphPad Prism 5 (GraphPad Software Inc., San Diego, CA, USA). Data of two groups were compared using Student’s t-tests, whereas multigroup data were compared using one-way analysis of variance (ANOVA); *p* < 0.05 was considered statistically significant.

## 3. Results

### 3.1. Establishment and Optimization of SMA-Cas14a1 Fluorescence Detection System Using ssDNA

**Figure 1:**
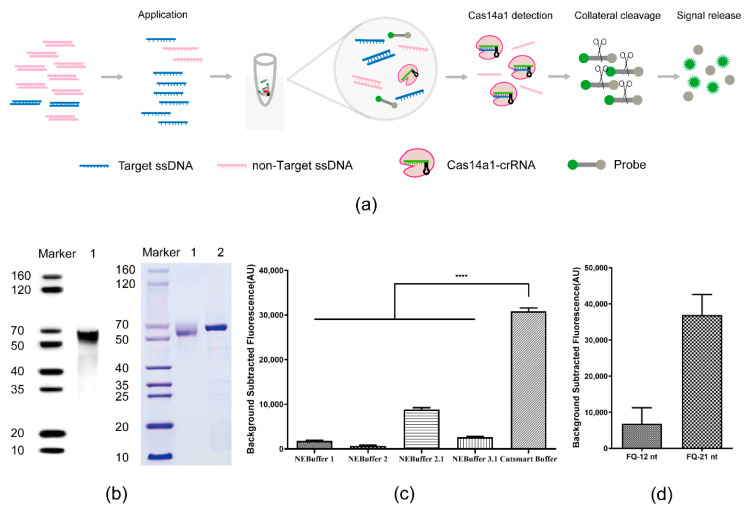
Cas14a1 Fluorescence Detection System. (**a**) Schematic of Cas14a1 fluorescence detection system. (**b**) Identification of Cas14a1 protein after purification: left, identification of Cas14a1 by Western blotting; right, Cas14a1 purity assessed using SDS-PAGE; BSA: control (lane 1). (**c**) Comparison of fluorescence signals obtained using different buffer candidates; ****p < 0.0001. (**d**) Comparison of fluorescence signals obtained using different lengths of FQ probe: FQ-12nt, length of FQ probe = 12 nt; FQ-21nt, length of FQ probe = 21 nt.

The scheme of the Cas14a1 fluorescence detection system is shown in Figure 1a. In this assay system, the targeted DNA is amplified, and the amplified targeted DNA triggers Cas14a1 activation after being recognized by the Cas14a1 endonuclease guided by the specific crRNA; the activated Cas14a1 then degrades the ssDNA-FQ probe through nonspecific trans-cleavage activity and yields a fluorescence signal.

First, Cas14a1 was expressed and purified (Figure 1b) and the crRNA was designed and transcribed in vitro. Next, Cas14a1 collateral cleavage activity was optimized using a series of buffers and measuring the fluorescence signals; the fluorescence signal was generated most effectively in the Cutsmart Buffer (Figure 1c). Moreover, in terms of the FQ probe length, FQ-21nt was more efficient than FQ-12nt in generating the signal (Figure 1d). Together, these results indicated that an appropriate and efficient platform for a sensitive biosensing assay was established.

### 3.2. Detection Performance of SMA-Cas14a1 Detection System Using gDNA Extracted from Cells

**Figure 2:**
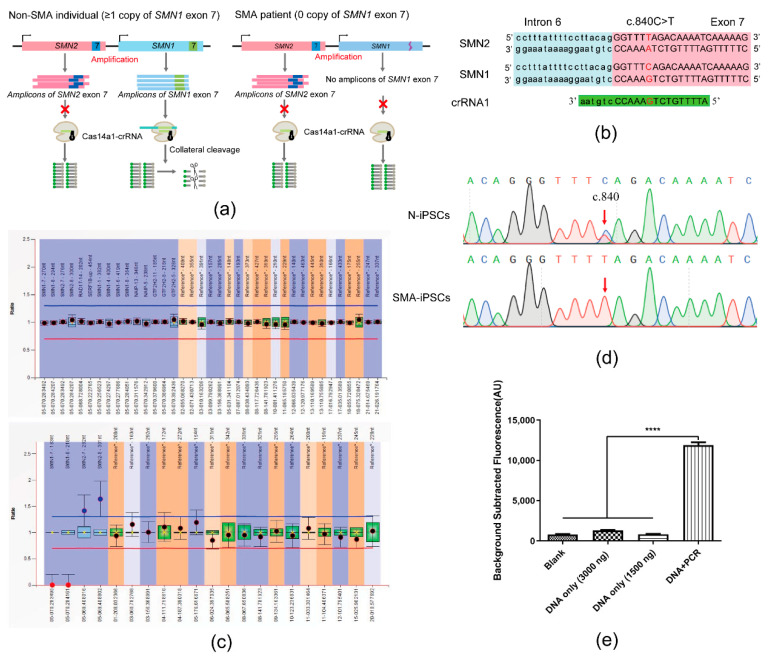
SMA-Cas14a1 Fluorescence Detection System. (**a**) Schematic illustration of SMA-Cas14a1 fluorescence detection system. The CRISPR RNA (crRNA) targeted exon 7 of *SMN1*. In non-SMA individuals, *SMN1* exon 7 triggers Cas14a1 activation after being recognized by the Cas14a1/crRNA complex, and activated Cas14a1 degrades the vicinal fluorescence probe nonspecifically, yielding fluorescence through acquired collateral cleavage activity. The Non-SMA individual included the SMA carrier with one copy of *SMN1* exon 7 and the normal individual with more than one copy of *SMN1* exon 7. However, SMA patients lack *SMN1* exon 7, which is required to activate Cas14a1, and thus no fluorescence is generated in this case. (**b**) Schematic representation of the position and sequence targeted by crRNA1. (**c**) MLPA results of N-iPSCs and SMA-iPSCs. (**d**) Sanger sequencing results of the PCR products of N-iPSCs and SMA-iPSCs using primers F1/R1. (**e**) Comparison of fluorescence in different groups: 3000 ng of DNA without amplification, 1500 ng of DNA without amplification, DNA amplified using PCR, and blank control. Data are means ± SEM; **** *p* < 0.0001.

**Figure 3:**
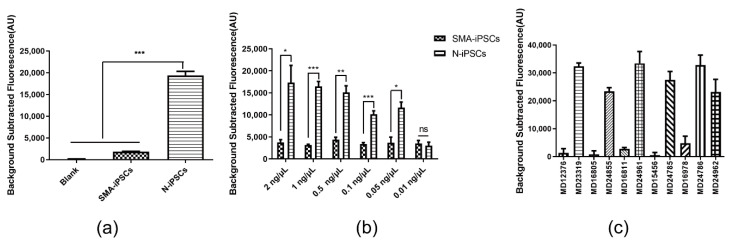
Detection Performance of Secondary PCR and SMA-Cas14a1 Assay. (**a**) Fluorescence signals of DNA from N-iPSCs and SMA-iPSCs amplified using secondary PCR. (**b**) Minimum detectable concentration of gDNA of SMA-Cas14a1 assay combined with secondary PCR. Data are means ± SEM; *** *p* < 0.005, ** *p* < 0.01, * *p* < 0.05; ns, *p* > 0.05. (**c**) Validation of SMA-Cas14a1 assay combined with secondary PCR by using gDNA from clinical samples. Data are means ± SEM.

Figure 2a illustrates the scheme of the SMA-Cas14a1 assay. The crRNA targeting *SMN1* was designed (Figure 2b) and transcribed in vitro, and gDNA was extracted from SMA-iPSCs and N-iPSCs for evaluating the detection performance of the SMA-Cas14a1 detection system. The SMA-iPSCs and N-iPSCs were detected using MLPA (Figure 2c). The gDNA samples were amplified using the primers F1/R1 and the PCR products were subjected to Sanger sequencing (Figure 2d), which revealed that the PCR products from SMA-iPSCs contained only one peak of ‘T’ at c.840, whereas the PCR products from N-iPSCs featured peaks of ‘C’ and ‘T’ at c.840. However, when the gDNA samples were used without PCR amplification, no clear fluorescence signals were detected and the samples could not be distinguished from each other (Figure 2e). Considering that Cas14a1 mainly binds and cleaves the target ssDNA, we used secondary PCR to obtain the ssDNA, and we measured a 10.58-fold difference in the fluorescence signal between N-iPSCs and SMA-iPSCs (Figure 3a), these results showed that N-iPSCs and SMA-iPSCs can be distinguished using SMA-Cas14a1 and secondary PCR.

### 3.3. Detection Performance and Application of Secondary PCR and SMA-Cas14a1 Assay with Clinical Samples

To determine the minimum gDNA concentration in samples from SMA patients and non-SMA individuals that can be distinguished using our assay, we tested serial dilutions of gDNA from N-iPSCs and SMA-iPSCs: The increase in fluorescence differed significantly between N-iPSCs and SMA-iPSCs when the DNA concentrations were >0.05 ng/μL (Figure 3b). When the molecular weight of the human genome (1.9 × 10^12^ g/mol, ~2.9 Gb) is used in calculations, the measured DNA concentration converts to ~26.3 aM. To evaluate the feasibility of applying secondary PCR combined with the SMA-Cas14a1 assay in the diagnosis of clinical samples, we analyzed gDNA isolated from the peripheral blood of 6 non-SMA individuals and 5 SMA patients (in whom SMA was diagnosed using MLPA). The results allowed the SMA patients to be notably distinguished from the non-SMA individuals, demonstrating the feasibility of using secondary PCR combined with the SMA-Cas14a1 assay for clinical diagnosis (Figure 3c).

### 3.4. Detection Performance of Asymmetric PCR Combined with SMA-Cas14a1 Assay

**Figure 4:**
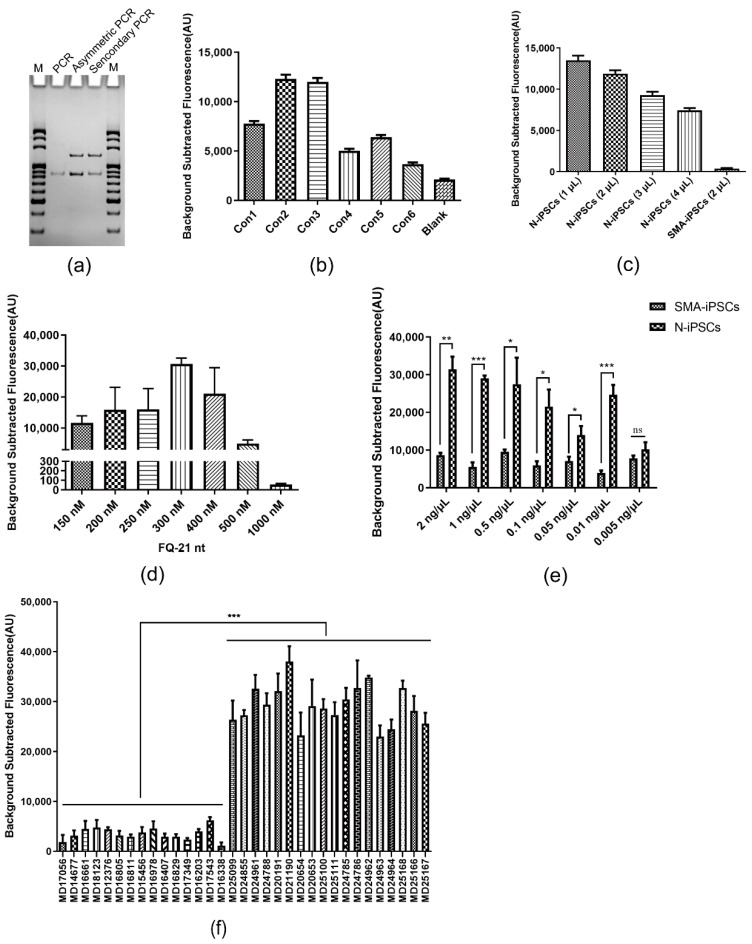
Detection Performance of Asymmetric PCR and SMA-Cas14a1 Assay. (**a**) Products of asymmetric PCR and secondary PCR were identified using electrophoresis. M, 20 bp DNA Ladder marker; PCR, double-stranded PCR product control. (**b**) Fluorescence signals obtained under different conditions of asymmetric PCR. Con1: 1 µM R1, with 40:1 ratio of F1 to R1; Con2: 1 µM R1, with 50:1 ratio of F1 to R1; Con3: 1 µM R1, with 60:1 ratio of F1 to R1; Con4: 2 µM R1, with 40:1 ratio of F1 to R1; Con5: 2 µM R1, with 45:1 ratio of F1 to R1; Con6: 2 µM R1, with 50:1 ratio of F1 to R1. (**c**) Fluorescence signals obtained using different amounts of asymmetric PCR product in the detection system. (**d**) Fluorescence signals obtained using different concentrations of FQ-21nt probe in the detection system. (**e**) Minimum detectable concentration of gDNA of SMA-Cas14a1 assay combined with asymmetric PCR. Data are means ± SEM; ***p < 0.005, **p < 0.01, *p < 0.05; ns, *p* > 0.05. (**f**) Validation of SMA-Cas14a1 assay combined with asymmetric PCR by using gDNA from clinical samples. Data are means ± SEM; ***p < 0.005.

To detect *SMN1* exon 7 deletion more simply and rapidly than in the previous assay, asymmetric PCR combined with the SMA-Cas14a1 assay was used for SMA detection. First, the product of asymmetric PCR was identified using electrophoresis, which revealed the presence of the target ssDNA similarly as secondary PCR did (Figure 4a). Second, the fluorescence signals were measured with the use of distinct concentrations of the primer R1 and varying ratios of F1 and R1. The strongest fluorescence was obtained when R1 was used at 1 µM and the ratio of F1 to R1 was 50:1 (Figure 4b). Third, different amounts of the asymmetric PCR product in the detection system of the SMA-Cas14a1 assay were evaluated based on the fluorescence signals, which revealed that the strongest fluorescence was obtained when 1 µL of the asymmetric PCR product was included in a detection volume of 20 µL (Figure 4c). Fourth, in assays for optimizing the FQ-21nt probe concentration, the strongest fluorescence was generated when the probe was used at 300 nM (Figure 4d). Fifth, the minimum detectable gDNA concentration was determined by testing serial dilutions of DNA from N-iPSCs and SMA-iPSCs; this revealed that the fluorescence increase differed significantly between N-iPSCs and SMA-iPSCs when the DNA concentrations were >0.01 ng/μL (Figure 4e), which converts to ~5.26 aM according to the molecular weight of the human genome. Lastly, we analyzed gDNA isolated from the peripheral blood of 18 non-SMA individuals and 15 SMA patients (in whom SMA was diagnosed using MLPA), with the goal being to assess the feasibility of using asymmetric PCR combined with the SMA-Cas14a1 assay. Our results agreed with the results of MLPA and qPCR, showing that the SMA patients could be clearly distinguished from the non-SMA individuals using asymmetric PCR combined with the SMA-Cas14a1 assay (Figure 4f).

## 4. Discussion

SMA—one of the most severe and common genetic diseases—leads to infant deaths and places substantial emotional and economic burden on the patients and society. A hot spot pathogenic mutation that causes SMA is homozygous deletion of *SMN1* exon 7. Recently, the United States Food and Drug Administration has approved Spinraza, Risdiplam and Onasemnogene abeparvovec for the treatment of SMA by countering the effect of exon 7 mutation through targeting of alternative splicing or gene therapy [6,7,8,9,10]. Thus, detection of the exon 7 deletion mutation of *SMN1* is of considerable social value and practical importance for the diagnosis and early treatment of SMA patients.

CRISPR/Cas-based nucleic acid detection provides a new option for genetic testing. However, to date, most of the genetic testing based on the collateral cleavage activity of Cas12, Cas13 and Cas14 has been focused on pathogen detection [25,26,27,28,29,30,31] and has seen little use in the detection of disease-causing mutations in human genetic diseases. As compared with Cas12, Cas14 not only shows a lack of PAM restriction and thus can be used in assays designed free of this requirement, Cas14 also exhibits higher recognition and discrimination of ssDNA sequences [22]. Zhang et al., introduced an artificial mismatch into the crRNA in their assay to avoid *SMN2* cross-reaction, and the results showed a 7.41-fold fluorescence difference between samples from non-SMA individuals and SMA patients, which was higher than the difference measured using the wild-type crRNA (2.26-fold) [20]. By comparison, this difference between non-SMA individuals and SMA patients reached 10.58-fold with the use of secondary PCR combined with the SMA-Cas14a1 assay here, which demonstrated the higher specificity and discrimination of Cas14a1 than Cas12a. In terms of the minimum detectable concentration of DNA with the two SMA-Cas assays, SMA-Cas12a used with a fluorescence probe reached a limit of 0.1 ng/µL (~52.6 aM), and the lowest DNA concentration detected was 526 aM when recombinase polymerase amplification was combined with the SMA-Cas12a-strip assay. In this study, the minimum DNA concentrations detected were ~26.3 aM with secondary PCR combined the SMA-Cas14a1 assay and ~5.26 aM (~3 copies/µL) with asymmetric PCR combined with the SMA-Cas14a1 assay, which hold great value for the detection of a small amount of DNA from dried blood spots, oral swabs, cell-free DNA and other specimens. Moreover, Cas14a1 has not been previously used for detecting disease-causing mutations in human genetic diseases. In addition, the CRISPR/Cas14a1 can be used in targeted sequencing with Oxford Nanopore Technologies (ONT), which employs the targeted cleavage of double stranded DNA with PAM restriction and targeted cleavage of single stranded DNA without PAM restriction with CRISPR/Cas14a1 to ligate adaptors for nanopore sequencing [32,33].

In terms of Cas14a1-based detection, the endonuclease can cleave the targeted ssDNA in the absence of a PAM sequence, and thus the approach used for obtaining this ssDNA is vital for nucleic acid detection by using CRISPR/Cas14a1. The secondary PCR step must be performed twice to obtain the targeted ssDNA. In 2018, Doudna et al., reported that the targeted ssDNA could be obtained through PCR amplification by using phosphorothioate-modified primers and then T7 exonuclease treatment [21]. In this study, the targeted ssDNA was amplified from gDNA by using asymmetric PCR, and the FQ probe was cleaved by activated Cas14a1 when the Cas14a1-crRNA complex specifically hybridized with the target ssDNA in vitro, which produced a fluorescence signal that could be detected and directly related to the amplified target. The target ssDNA amplified using asymmetric PCR can be directly used for detection without the requirement of other steps, which substantially shortens the assay time [34].

## 5. Conclusions

We developed a nucleic acid diagnostic platform for detecting the principal disease-causing mutation in SMA by exploiting the collateral cleavage activity of CRISPR/Cas14a1 and asymmetric PCR. This diagnostic platform enable specific, sensitive, and accurate detection of the deletion of *SMN1* exon 7, which holds significant implications for the diagnosis and early treatment of SMA. Given the absence of a PAM-sequence restriction, this system is easy to use, adaptable, and scalable for broadly detecting disease-causing mutations in human genetic diseases.

## Data Availability

All data supporting the reported result in this study can be found in the Supplementary Materials.

## Supplementary Materials

The following supporting information can be downloaded at: https://www.mdpi.com/article/10.3390/bios12050268/s1, Figure S1: Schematic representation of the locations of PCR primers and crRNA seed region. The primers F1 and R1 used for PCR in this study were indicated by green arrows. The *SMN1* c.840C>T was indicated in bold and red. The crRNA seed region was indicated with a red line. The exon 7 of *SMN1* was in gray shadow. The partial intron 6 of *SMN1* was in blue and underlined by a blue line. The partial intron 7 of *SMN1* was in orange and underlined by a orange line; Table S1: Detailed sequences of primers, crRNAs, and probes in this study.
