# Supplementary material for: Cas14a1-Mediated Nucleic Acid Diagnostics for Spinal Muscular Atrophy
Source: Biosensors (Basel). 2022 Apr 23;12(5):268. doi: 10.3390/bios12050268 (PMC9138763; doi:10.3390/bios12050268)
Supplement: Supplementary file 1 [file biosensors-12-00268-s001.zip › biosensors-1673498-supplementary.pdf]

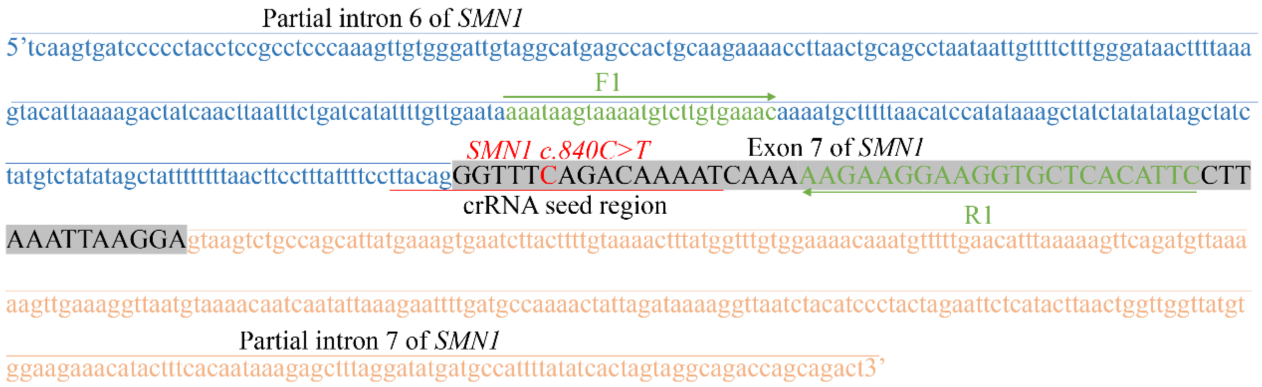

Figure S1. Schematic representation of the locations of PCR primers and crRNA seed region. The primers F1 and R1 used for PCR in this study were indicated by green arrows. The *SMN1* c.840C>T was indicated in bold and red. The crRNA seed region was indicated with a red line. The exon 7 of *SMN1* was in gray shadow. The partial intron 6 of *SMN1* was in blue and underlined by a blue line. The partial intron 7 of *SMN1* was in orange and underlined by a orange line.

Table S1. Detailed sequences of primers, crRNAs and probes used in this study.

**name.**	**Sequence (5′ to 3′)**
crRNA	taatacgactcactataggTTCACTGATAAAGTGGAGAACCGCTTCACCAAAA-<br>GCTGTCCCTTAGGGGATTAGAACTTGAGTGAAGGTGGGCTGCTTGCATCAGCCTAAT<br>GTCGAGAAGTGCTTTCTTCGGAAAGTAACCCTCGAAACAAATTCATTTgaaa-<br>GAATGAAGGAATGCAACATTTGTCTGAAACCctgtaa
crRNA-F	AAATAAGTAAAATGTCTTGTGAAAC
crRNA-SMN1-R	GAATGTGAGCACCTTCCTTCTT
F1	AAATAAGTAAAATGTCTTGTGAAAC
R1	GAATGTGAGCACCTTCCTTCTT
FQ-21nt	5'FAM-TTTTTTTTTTTTTTTTTTTTT-3'BHQ-X
FQ-12nt	5'FAM-TTTTTTTTTTTT-3'BHQ-X
SMN1-ssDNA	tttaacttcctttattttccttacagGGTTTCAGACAAAATCAAAAAGAAGGAAGGTGC
